# Comparative Analysis of Repetitive DNA between the Main Vectors of Chagas Disease: *Triatoma infestans* and *Rhodnius prolixus*

**DOI:** 10.3390/ijms19051277

**Published:** 2018-04-24

**Authors:** Sebastián Pita, Pablo Mora, Jesús Vela, Teresa Palomeque, Antonio Sánchez, Francisco Panzera, Pedro Lorite

**Affiliations:** 1 Evolutionary Genetic Section, Faculty of Science, University of the Republic, Iguá 4225, Montevideo 11400, Uruguay; spita@fcien.edu.uy; 2 Department of Experimental Biology, Genetics , University of Jaén, Paraje Las Lagunillas s/n., 23071 Jaén, Spain; pmora@ujaen.es (P.M); jvela@ujaen.es (J.V.); tpalome@ujaen.es (T.P.); abaca@ujaen.es (A.S)

**Keywords:** Chagas disease vectors, *Triatoma infestans*, *Rhodnius prolixus*, satellite DNA, genomic evolution, fluorescence in situ hybridization

## Abstract

Chagas disease or American trypanosomiasis affects six to seven million people worldwide, mostly in Latin America. This disease is transmitted by hematophagous insects known as “kissing bugs” (Hemiptera, Triatominae), with *Triatoma infestans* and *Rhodnius prolixus* being the two most important vector species. Despite the fact that both species present the same diploid chromosome number (2*n* = 22), they have remarkable differences in their total DNA content, chromosome structure and genome organization. Variations in the DNA genome size are expected to be due to differences in the amount of repetitive DNA sequences. The *T. infestans* genome-wide analysis revealed the existence of 42 satellite DNA families. BLAST searches of these sequences against the *R. prolixus* genome assembly revealed that only four of these satellite DNA families are shared between both species, suggesting a great differentiation between the *Triatoma* and *Rhodnius* genomes. Fluorescence in situ hybridization (FISH) location of these repetitive DNAs in both species showed that they are dispersed on the euchromatic regions of all autosomes and the X chromosome. Regarding the Y chromosome, these common satellite DNAs are absent in *T. infestans* but they are present in the *R. prolixus* Y chromosome. These results support a different origin and/or evolution in the Y chromosome of both species.

## 1. Introduction

Chagas disease is an anthropozoonotic illness caused by the protozoan parasite *Trypanosoma cruzi*. It affects six to seven million people worldwide, mostly in Latin America but it is increasingly being detected in USA, Canada, and many European countries [1,2,3]. In Latin America, this disease is transmitted by hemipteran insects of the subfamily Triatominae, known as “kissing bugs”. This group included 151 species, being *Triatoma infestans* and *Rhodnius prolixus* the two major vector species, both by their extensive geographic distribution and their high effectiveness in transmitting the parasite to human hosts [4]. Although in recent years their geographical distributions have been substantially reduced due to control campaigns, they continue to represent a serious threat to human health [5,6]. In Colombia and Venezuela, *R. prolixus* is the primary vector, while in large regions of Bolivia, Paraguay and Argentina *T. infestans* is responsible for more than 30% of new cases that occur by vector transmission throughout Latin America [7]. Within *T. infestans*, two main lineages, named Andean and non-Andean, are clearly differentiated by genetic [8,9] and phenetic characteristics [10,11], with dissimilar geographic distribution and epidemiological capacities [12].

*Triatoma infestans* and *R. prolixus* are included in two different tribes, with marked morphological differentiation: Triatomini tribe for *T. infestans* and Rhodniini tribe for *R. prolixus* [13]. In spite that both species present the same diploid chromosome number (2*n* = 22), they have striking differences in their total DNA content, chromosome structure and genome organization. *Rhodnius prolixus* has a haploid genome size of 733 Mb without autosomal C-heterochromatic regions, and the Y chromosome entirely heterochromatic [14]. The *T. infestans* genome is more than twice that *R. prolixus.* The genome size of *T. infestans* is variable between Andean and non-Andean lineages (1936 Mb and 1487 Mb respectively), with significant differences in the number of autosomes with heterochromatic regions, and the Y heterochromatic in both lineages [8,12]. Recently, genome wide analysis using RepeatExplorer with Illumina (San Diego, CA, USA) reads showed that satellite DNA (satDNA) sequences are the major component of repetitive DNA (33% and 25% in Andean and non-Andean genomes respectively) [15]. This study determined that the variation in the number of copies of four satDNA families located on the heterochromatic regions significantly contributed to the genomic DNA differentiation between both *T. infestans* lineages. Moreover, several satDNA families were unexpectedly detected on the euchromatic regions of the autosomes and the X chromosome. All transposable elements (TEs) represented a minor fraction within the genome, being around 6% in both lineages.

*Rhodnius prolixus* represents the first assembled genome of a non-dipteran insect vector of a human parasitic disease [16]. Through the methodology of whole-genome shotgun sequencing using Sanger and others technologies 95% of the genome was assembled, with an 8× genome coverage. A total of 15,456 protein-coding genes and 738 RNA genes were identified, including nine Y-linked genes. Within the repetitive fraction, TEs comprised about 5.6% of the total genome. Other repeated sequences such as satDNA were not analyzed, probably because it would represent a very minor fraction of the genome or perhaps due to the difficulty posed by its assembly.

In a first attempt to reveal the identity of the *R. prolixus* satDNA fraction, we compared the satDNA fraction of the *T. infestans* genome [15] with the assembled genome of *R. prolixus* [16]. Furthermore, we determined the chromosome localization of these common satDNA families using fluorescence in situ hybridization (FISH) in both species.

## 2. Results

### 2.1. Characterization of Triatoma Infestans Satellite DNA Families in the Rhodnius Prolixus Genome

The 42 satDNA families previously isolated in *T. infestans* [15] were searched in the *R. prolixus* genome assembly. As results, four *T. infestans* satDNA families were found in *R. prolixus* (TinfSat04-1000, TinfSat12-84, TinfSat15-99 and TinfSat33-372).

#### 2.1.1. TinfSat04-1000

**Figure 1:**
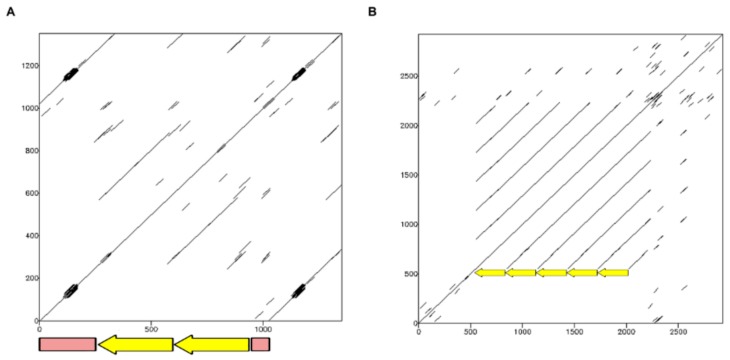
(**A**) Dotplot of one of the clusters for TinfSat04-1000 obtained in the RepeatExplorer analysis of the *T. infestans* genome, showing the presence of two internal repeats of about 300 bp (arrows) and an unrelated sequence of about 400 bp (box) within the 1 kb repeat; (**B**) Dotplot of the internal region of a *R. prolixus* superconting that shows similarity with the TinfSat04-1000 satDNA of *T. infestans*, revealing repeats with a periodicity of about 300 bp (arrows).

In *T. infestans*, TinfSat04-1000 has a repeat unit of 1000 bp [15]. A more detailed analysis of this sequence has revealed the existence of two internal repeats of about 300 bp, with a similarity of 88.4% (Figure 1A). Sequence analysis of the *R. prolixus* scaffolds showed the existence of tandem arrays of sequences with similarity with TinfSat04-1000. The repeat unit of these sequences is 293 bp in length (Figure 1B and Figure S1). The similarity of the two repeats of *T. infestans* with the consensus sequence of the *R. prolixus* satDNA is 69.2% and 73.1%, respectively (Figure S2). Primers for polymerase chain reaction (PCR) were designed to check if the 1000 bp satDNA was a RepeatExplorer artifact (Figure S3). Analysis of the PCR products obtained demonstrated that this repetitive family has a repeat unit of 1000 bp in the *T. infestans* genome (Figure S3). No amplification was observed when the same primers were used on *R. prolixus*. Our PCR results were consistent with in silico analyses, demonstrating that only the 300 bp satDNA is present in *R. prolixus* genome. Finally, a search on CENSOR using the 400 bp fragment of the *T. infestans* satellite as query, indicated that this sequence has stretches with similarity (72% to 89%) with *Helitron* TEs from *R. prolixus*.

#### 2.1.2. TinfSat12-84

Several scaffolds were retrieved as hits in *R. prolixus*, and several copies of this satDNA were located on each scaffold. The length of these repeats was similar to that found in the TinfSat12-84 of *T. infestans* (about 84 bp). The alignment showed that TinfSat12-84 is very variable in *R. prolixus*. The similarity among the 34 complete repeats retrieved from the analyzed scaffolds ranged between 50% and 99%. Variations among the sequences are mainly due to nucleotide substitutions and small insertion and deletions of one to three nucleotides (Figure S4). The similarity between the repeats retrieved from the *R. prolixus* genome and the consensus sequence of the *T. infestans* TinfSat12-84 satDNA ranges between 59% and 84%.

#### 2.1.3. TinfSat15-99

Only two *R. prolixus* scaffolds containing five repeats were recovered, with slight differences between monomers, as depicted in the Figure S5. Upon comparison with the consensus sequence of *T. infestans*, a 11 bp deletion was identified in some monomers from *R. prolixus* that is absent in *T. infestans*, as well as a 5 bp insertion that also appears in some *T. infestans* monomers. The similarity between the repeats retrieved from the *R. prolixus* genome and the consensus sequence of the *T. infestans* TinfSat15-99 satDNA ranges between 93% and 97%.

#### 2.1.4. TinfSat33-372

**Figure 2:**
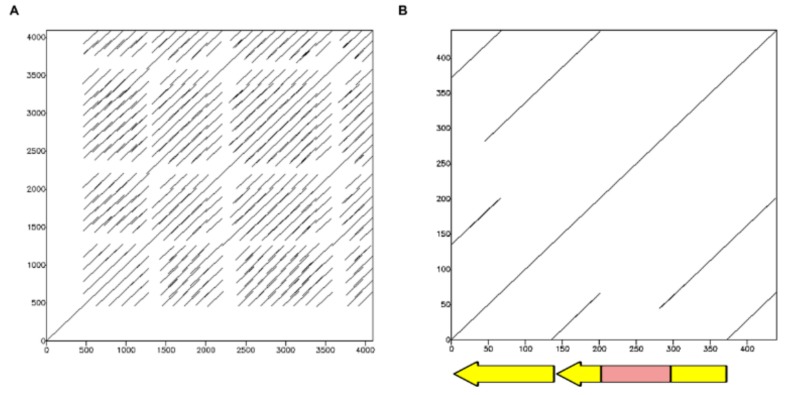
(**A**) Dotplot of a region of a *R. prolixus* superconting that shows similarity with the Tinf33-372 satDNA of *T. infestans*. The superconting contains several tandems of repeats of about 135 bp in length; (**B**) Dotplot of one of the cluster for TinfSat33-372 obtained in the RepeatExplorer analysis of the *T. infestans* genome. The 372 bp repeat contains two internal repeats of about 135 bp (arrows) and an unrelated sequence of about 100 bp (box).

The consensus sequence of the *T. infestans* TinfSat33-372 satDNA was used as a query and several *R. prolixus* genome scaffolds were retrieved. These scaffolds contain several monomers with 136 bp in length (Figure 2A and Figure S6). TinfSat33-372 in *T. infestans* has a complex structure, in which the repeat unit of 372 bp contains two internal repeats of 135 and 136 bp, one of them interrupted by an unrelated sequence of 101 bp (Figure 2B). Searches using the latter sequence in the databases revealed no significant similarity to any deposited sequences; while the 136 bp sequence presented similarity with both *T. infestans* internal repeats (Figure S7).

### 2.2. C-Banding and Chromosome Location of the Satellite DNA Families Shared between Triatoma infestans and Rhodnius prolixus

**Figure 3:**
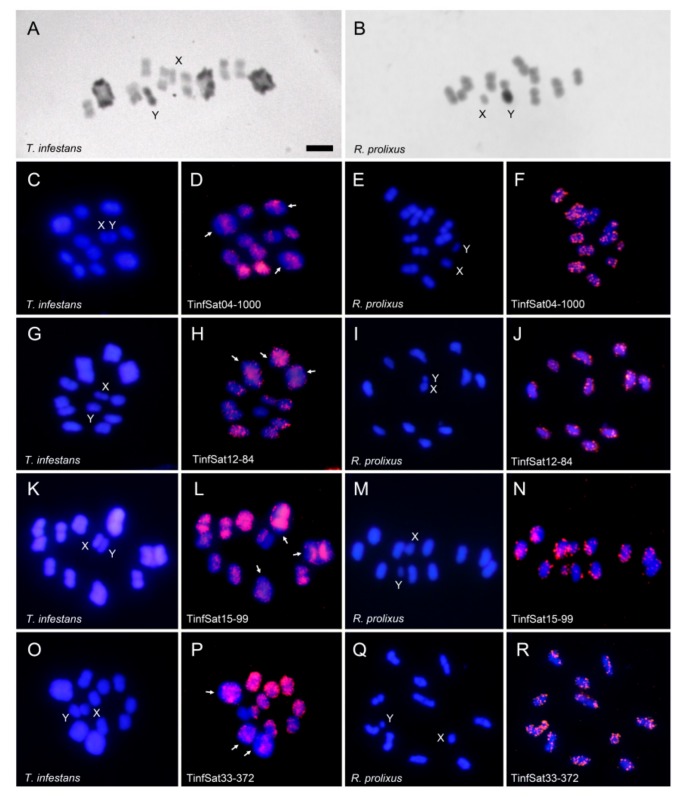
C-banding and chromosome location of the satDNA families in male meiotic chromosomes (first meiotic metaphases) of *Triatoma infestans* and *Rhodnius prolixus*. C-banding in *T. infestans* (**A**) and *R. prolixus* (**B**); (**C**–**R**) First and third columns DAPI staining (blue) and second and forth columns FISH with the different satDNA probes (pink). Arrows in D, H, L and P indicate some of the heterochromatic regions lacking hybridization signals on the three largest bivalents of *T. infestans*. Bar = 5 μm.

The distribution of heterochromatin is different between both species, as revealed by C-banding. In the analyzed *T. infestans* individuals (non-Andean lineage) heterochromatic regions are present on the three largest autosomal bivalents as well as on the Y chromosome (Figure 3A). In *R. prolixus* the presence of heterochromatin is restricted to the Y chromosome (Figure 3B).

In a previous study the main eleven satDNAs in the *T. infestans* genome were located by FISH, including TinfSat04-1000 [15]. This satDNA is dispersed on the euchromatic regions of the autosomes and the X chromosome (Figure 3D). FISH with the other three shared satDNA families showed that they are also located on euchromatic regions. Heterochromatic Y chromosome lacked any hybridization signals (Figure 3H,L,P). In *R. prolixus*, as well as in *T. infestans*, hybridization signals are dispersed on all euchromatic autosomes as well as on the euchromatic X chromosome. The four satDNA families also generate hybridization signals on the heterochromatic Y chromosome of *R. prolixus* (Figure 3F,J,N,R).

## 3. Discussion

The analysis of the *T. infestans* genome revealed the presence of at least 42 satellite DNA families [15]. These repetitive DNAs are the main component of the *T. infestans* genome, representing 33% and 25% of the genomic DNA in Andean and non-Andean lineages, respectively. Most satDNA is located on the heterochromatic regions. However, there are numerous satDNA families on the euchromatic regions [15]. The FISH analyses carried out here with other three new satDNAs in *T. infestans* revealed that these satDNAs are also located on euchromatic regions of the autosomes and the X chromosome.

**Figure 4:**
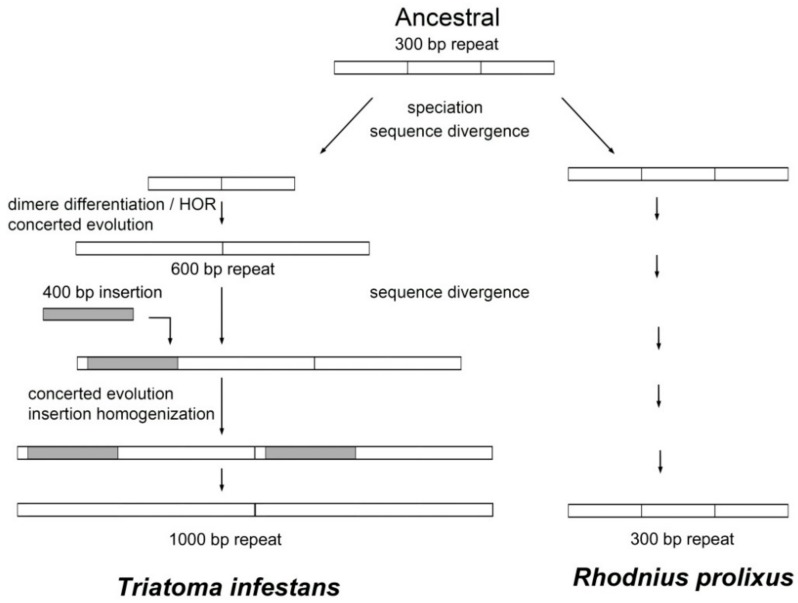
Hypothetical pathway of the TinfSat04-1000 satellite DNA in *T. infestans* and *R. prolixus*. The ancestral situation would be the existence of a satDNA family with a repeat unit of 300 bp. This organization could be retained in the *R. prolixus* genome. The evolution of this repetitive DNA would be more complex in *T. infestans.* The 300 bp ancestral satDNA could have generated a new satDNA family with a repeat unit of 1000 bp. First, a HOR would have formed from a dimer, which would generate a 600 bp satDNA. Second, an insertion of about 400 bp would have taken place within this new repetition. Concerted evolution would spread this insertion resulting in a new tandem repeat of 1000 bp.

**Figure 5:**
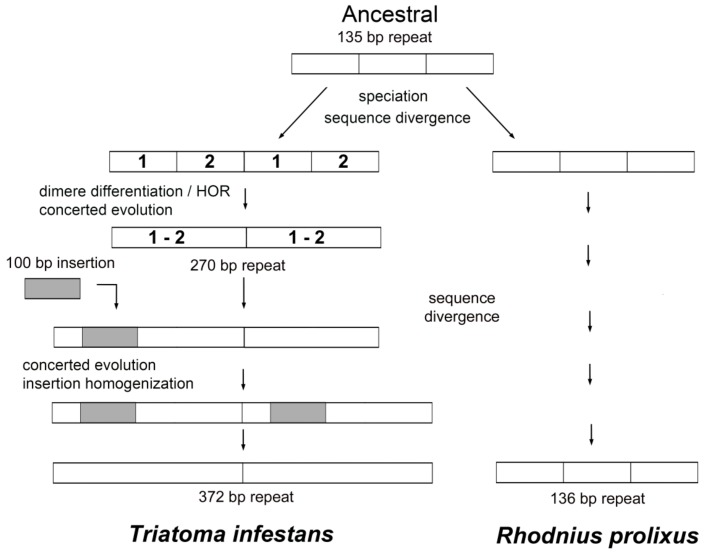
Hypothetical pathway of the TinfSat33-372 satellite DNA in *T. infestans* and *R. prolixus*. The ancestral situation would be the existence of a satDNA family with a repeat unit of 135 bp. This organization is conserved in the *R. prolixus* genome with a repeat unit of 136 bp. The repeat unit of 372 bp of *T. infestans* would has been generated from the ancestral satDNA by HOR formation and an insertion of a sequence of about 100 bp.

BLAST search results showed that only four satDNA families of the 42 isolated in the *T. infestans* genome had some similarity with the *R. prolixus* assembled genome. The existence of shared satDNA families suggests a common origin of both species and therefore that these repetitive DNAs should be present in the common ancestor. However, 38 of the 42 satDNA families identified in *T. infestans* are not present in *R. prolixus*. SatDNA analysis indicates that the differentiation between both species involves an important genome remodeling, at least in relation to repetitive DNA sequences. It has been claimed that the estimated divergence between *T. infestans* and *R. prolixus* was dated at 40–35 million years ago [17,18]. Probably, the differentiation between both species involves the presence or absence of different satDNA families. In some shared satDNAs the repeat units have intense modifications, such as TinfSat04-1000 or TinfSat33-372. The repeat unit of TinfSat04-1000 in *T. infestans* is 1000 bp in length but the satDNA with similarity in *R. prolixus* has a repeat unit of 300 bp. This allowed us to hypothesize about the evolution of this satDNA family in the triatomines. Whereas in *R. prolixus* genome the ancestral 300 bp motif could have been preserved, in *T. infestans* this motif suffered a very distinct path. First, a higher order repeat (HOR) structure might have formed, and after a 400 bp fragment was inserted and spread within the satDNA, resulting in a new repeat unit of 1000 bp (Figure 4). We draw a similar hypothetic scenario for the TinfSat33-372 evolution. While in *R. prolixus* the ancestral 135 bp tandem array is conserved, in *T. infestans* genome a new repeat unit of 372 bp is generated by a HOR formation and a 101 bp fragment insertion (Figure 5). HOR formation has been described for other satDNA families in *T. infestans* [15], suggesting that the trend in structure is common in Triatominae satDNA. The 400 bp fragment of TinfSat04-1000 showed homology with *Helitron* TEs. The process in which satDNA sequences and TEs interact to create new repetitive DNA has been reported in several genomes [19]. Sometimes satDNA repeats generated by TE sequences are localized on a restricted locus [19]. It will not be the case for TinfSat04-1000, which is distributed throughout the genome. Interestingly, sequences with similarity to *Polinton* TEs have been found adjacent to arrays of other satDNA families of *T. infestans* [20]. Moreover, the authors suggest that *Polinton* TEs could providing the mechanism by which these satDNA repeats could propagate in the genome [20], as has been suggested for other insect satDNA [21]. Hence, it is possible that other class II elements could contribute to satDNA architecture.

Unlike the two satDNA families discussed above, TinfSat12-84 and TinfSat15-99 showed similar structure in both genomes. Differences between *T. infestans* and *R. prolixus* satellite repeats were only due to a few point mutations as well as short insertion/deletions that could be present in both genomes. Hence, both satDNAs could be highly conserved within Triatominae. It is unclear why some satDNAs are conserved for long periods of time while others evolve rapidly [22,23]. It has been hypothesized that a possible function of the satDNA may act as a constraint for its evolution [23]. It is widely assumed that satDNAs have a relevant function in the chromosome organization, pairing and segregation [24]. The evidence of transcription for many satDNAs reinforces its possible role in the chromosome function [24,25]. Functional centromeric domain is localized into the pericentromeric heterochromatin in monocentric chromosomes [26]. Triatominae species, as other hemipteran insects, are characterized by the presence of holocentric chromosomes which lack a located centromere, so that the centromeric function is distributed along the length of the chromosome. FISH assays showed that in both species several satDNA families are dispersed throughout all the euchromatin. So, a potential function could not be discarded despite its location since it has been proposed that dispersed satDNAs could be an important centromere determinant in species with holocentric chromosomes [26]. Future analyzes will be necessary to determine if this satDNA really has some function in Triatominae genomes.

One of the most obvious differences between the hybridization patterns in both species is the unequal distribution of the four satDNA families on the Y chromosome. Differences between the Y chromosomes in species of the tribe Triatomini and the tribe Rhodniini were previously reported. In Triatomini, the Y chromosome is mainly composed by A+T rich repeated DNA sequences [20,27]. Genomic in situ hybridization (GISH) analyses on fifteen Triatomini species from four different genera revealed that these repetitive sequences were highly conserved [28,29]. At least two satDNAs are present on the *T. infestans* Y chromosome, TinfSat01-33 and (GATA)_n_, but only (GATA)_n_ repeats are common to all Triatomini species [15]. The Y chromosome sequence conservation in species-rich insect groups is uncommon, so, its conservation in the Triatomini tribe probably represents an ancestral character in this group, as previously suggested by GISH analyses [28,29]. In Rhodniini, the heterochromatic Y chromosome is constituted by other types of DNA sequences that are not revealed by GISH (using *Triatoma* species probes) or fluorescence-banding analyses [27,28]. Our results provide new data about the different composition of the Y chromosomes between both species. The four shared satDNAs families are located on the *R. prolixus* Y chromosome but absent in the *T. infestans* Y chromosome. As above indicated, two satDNAs were located on the *T. infestans* Y chromosome; TinfSat01-33 and (GATA)n repeats. TinfSat01-33 was not recovered in the *R. prolixus* genome and FISH with a GATA probe resulted in a lack of hybridization signals on the *R. prolixus* Y chromosome. Moreover, a great differentiation among the X chromosomes from both tribes was also showed by chromosome painting employing X chromosome specific probes [30].

In conclusion, data indicate that the differentiation between Triatomine species has been accompanied by severe changes in their repetitive DNAs. The role of satDNA in the speciation processes it is a topic that has been under discussion for a long time. Several mechanisms have been proposed by which the satDNA could disrupt chromosome pairing in hybrids, acting like a reproductive barrier [23,31]. As a consequence, differences in the satDNA could reduce the fitness of the hybrids between sibling species. More studies will be necessary to determine if satDNA differentiation is the cause and/or the consequence of the speciation processes.

## 4. Materials and Methods

### 4.1. Bioinformatic Analyses

SatDNA families described for *T. infestans* were used as query against *R. prolixus* genome -assembly RproC3, available on https://www.vectorbase.org/organisms/rhodnius-prolixus/cdc/rproc3. A Basic Local Alignment Search Tool (BLAST) analysis was used to retrieve the shared satDNA families between both genomes. All hits were analyzed for each family in order to determine the structure of those satDNA families on *R. prolixus* genome. First, scaffolds containing BLAST hits were used to look for putative satDNA sequences. Determination of the repeat units was done using a dotplot tool (EMBOSS Dotmatcher, available on: http://emboss.bioinformatics.nl/cgi-bin/emboss/dotmatcher). Alignments of the monomers were used to determine the consensus sequence and the satellite variability within *R. prolixus* and *T. infestans*. For this purpose, several EMBOSS tools were used (available on: http://www.bioinformatics.nl/emboss-explorer/). The evolutionary divergence between sequences was estimated using the p-distance model in the program MEGA version 6 [32]. Comparative analysis with other repetitive sequences was carried out using CENSOR database (available on: http://www.girinst.org/censor/index.php).

### 4.2. Insects and DNA Extraction

*Triatoma infestans* and *R. prolixus* DNA was extracted from legs of adult male individuals from Argentina (Pampa Ávila, Chaco) (non-Andean lineage) and Colombia (Magdalena), respectively, following the NucleoSpin Tissue kit (Macherey-Nagel Co., Düren, Germany) instructions.

### 4.3. PCR Amplifications

PCR primers Tinf-CL2Uy-F (5′ GATATCGAAAATTTGACACG) and Tinf-CL2Uy-R1 (5′ ATGTATGTGAACAGCATAGC were designed to test the TinfSat04-1000 organization in both species. Conditions for the PCR assay consisted in a first denaturalization step at 92 °C for 90 s, 35 cycles of 92 °C for 20 s, 51 °C for 1 min, 72 °C for 2 min, and a last step of 72 °C for 5 min. Reactions were set up in a 25 μL mixture containing 100 ng of genomic DNA, 0.5 mM dNTPs, 50 pmol of each primer and 1 U of Taq polymerase. PCR results were analyzed by electrophoresis in 1% (*m*/*v*) agarose gel.

### 4.4. Chromosome Preparations, C-Banding and FISH

Meiotic chromosome preparations for C-banding and FISH analyses were obtained from male gonads. Individuals used here were the same as those used for DNA purifications. Testes were removed from living adult insects, fixed in an ethanol-glacial acetic acid mixture (3:1) and stored at −20 °C. Squashes were made in a 50% acetic acid drop, coverslips were removed after freezing in liquid nitrogen and the slides were air dried and then stored at 4 °C. C-banding was performed as previously described [8]. FISH probes were developed for each satDNA family. For TinfSat04-1000, we used the same probe as in Pita et al. [15]. For TinfSat12-84, TinfSat15-99 and TinfSat33-372, one oligonucleotide based on the most conserved regions was directly labeled with biotin-16-dUTP using terminal transferase (Roche, Mannheim, Germany) and following the instructions of the supplier. These were: TinfSat12-84-F: 5′ATATGCGAACACATACAGGCGAGAAGCCrT; TinfSat15-99-F: 5′ACCrTGCAACATGACATGTCTCAACATGTT; TinfSat33-372-F; 5′CkCGTTTGTGCCGGCGATTCACCAAATTTT. FISH were carried out as described by Palomeque et al. [33]. Hybridization solutions were prepared to a final concentration of 200 pmol probe/mL in 50% formamide. Hybridization was conducted at 37 °C overnight. Fluorescence immunological detection was performed using the avidin-FITC/anti-avidin-biotin system with two amplification rounds. Slides were mounted with Vectashield (Vector, Burlingame, CA, USA). DAPI in the antifade solution was used to counterstain chromosomes. The hybridized chromosomes were observed and photographed using a BX51 Olympus (Tokyo, Japan) fluorescence microscope equipped with a CCD camera (Olympus DP70) and merged using the Olympus DPManager software. Hybridization pattern for each species was determined by the chromosomal analyses of at least twenty metaphases.

## Supplementary Materials

Supplementary materials can be found at http://www.mdpi.com/1422-0067/19/5/1277/s1.

## Abbreviations

DAPI	4′,6-Diamidine-2′-phenylindole
FISH	Fluorescence in situ hybridization
FITC	Fluorescein-5-isothiocyanate
GISH	Genomic in situ hybridization
HOR	Higher order repeat
satDNA	Satellite DNA
TE	Transposable element

## References

[1] Gascon J., Bern C., Pinazo M.J. (2010). Chagas disease in Spain, the United States and other non-endemic countries. Acta Trop..

[2] Schmunis G.A., Yadon Z.E. (2010). Chagas disease: A Latin American health problem becoming a world health problem. Acta Trop..

[3] World Health Organization (2017). Chagas Disease (American Trypanosomiasis). http://www.who.int/mediacentre/factsheets/fs340/en/index.html.

[4] Justi S.A., Galvão C. (2017). The evolutionary origin of diversity in Chagas disease vectors. Trends Parasitol..

[5] Schofield C.J., Jannin J., Salvatella R. (2006). The future of Chagas disease control. Trends Parasitol..

[6] Hashimoto K., Schofield C.J. (2012). Elimination of *Rhodnius prolixus* in Central America. Parasit. Vectors.

[7] World Health Organization (2015). Chagas disease in Latin America: An epidemiological update based on 2010 estimates. Wkly. Epidemiol. Rec..

[8] Panzera F., Dujardin J.P., Nicolini P., Caraccio M.N., Rose V., Tellez T., Bermúdez H., Bargues M.D., Mas-Coma S., O’Connor J.E. (2004). Genomic changes of Chagas disease vector, South America. Emerg. Infect. Dis..

[9] Bargues M.D., Klisiowicz D.R., Panzera F., Noireau F., Marcilla A., Pérez R., O’Connor J.E., Galvão C., Jurberg J., Carcavallo R.U. (2006). Origin and phylogeography of the Chagas disease main vector *Triatoma infestans* based on nuclear rDNA sequences and genome size. Infect. Genet. Evol..

[10] Hernández L., Abrahan L., Moreno M., Gorla D., Catalá S. (2008). Phenotypic variability associated to genomic changes in the main vector of Chagas disease in the southern cone of South America. Acta Trop..

[11] Calderón-Fernández G.M., Girotti J.R., Juárez M.P. (2012). Cuticular hydrocarbon pattern as a chemotaxonomy marker to assess intraspecific variability in *Triatoma infestans*, a major vector of Chagas’ disease. Med. Vet. Entomol..

[12] Panzera F., Ferreiro M.J., Pita S., Calleros L., Pérez R., Basmadjián Y., Guevara Y., Breniére S.F., Panzera Y. (2014). Evolutionary and dispersal history of *Triatoma infestans*, main vector of Chagas disease, by chromosomal markers. Infect. Genet. Evol..

[13] Lent H., Wygodzinsky P. (1979). Revision of the Triatominae (Hemiptera, Reduviidae) and their significance as vector of Chagas disease. Bull. Am. Mus. Nat. Hist..

[14] Panzera F., Pérez R., Panzera Y., Ferrandis I., Ferreiro M.J., Calleros L. (2010). Cytogenetics and genome evolution in the subfamily Triatominae (Hemiptera, Reduviidae). Cytogenet. Genome Res..

[15] Pita S., Panzera F., Mora P., Vela J., Cuadrado A., Sánchez A., Palomeque T., Lorite P. (2017). Comparative repeatome analysis on *Triatoma infestans* Andean and Non-Andean lineages, main vector of Chagas disease. PLoS ONE.

[16] Mesquita R.D., Vionette-Amaral R.J., Lowenberger C., Rivera-Pomar R., Monteiro F.A., Minx P., Spieth J., Carvalho A.B., Panzera F., Lawson D. (2015). Genome of *Rhodnius prolixus*, an insect vector of Chagas disease, reveals unique adaptations to hematophagy and parasite infection. Proc. Natl. Acad. Sci. USA.

[17] Hwang W.S., Weirauch C. (2012). Evolutionary history of assassin bugs (Insecta: Hemiptera: Reduviidae): Insights from divergence dating and ancestral state reconstruction. PLoS ONE.

[18] Justi S.A., Galvão C., Schrago C.G. (2016). Geological changes of the Americas and their influence on the diversification of the Neotropical kissing bugs (Hemiptera: Reduviidae: Triatominae). PLoS Negl. Trop. Dis..

[19] Mestrović N., Mravinac B., Pavlek M., Vojvoda-Zeljko T., Satović E., Plohl M. (2015). Structural and functional liaisons between transposable elements and satellite DNAs. Chromosome Res..

[20] Bardella V.B., Aristeu J.A., Vanzela A.L.L. (2014). Origin and distribution of AT-rich repetitive DNA families in *Triatoma infestans* (Heteroptera). Infect. Genet. Evol..

[21] Palomeque T., Carrillo J.A., Muñoz-López M., Lorite P. (2006). Detection of a *mariner*-like element and a miniature inverted-repeat transposable element (MITE) associated with the heterochromatin from ants of the genus *Messor* and their possible involvement for satellite DNA evolution. Gene.

[22] Palomeque T., Lorite P. (2008). Satellite DNA in insects: A review. Heredity.

[23] Plohl M., Meštrović N., Mravinac B. (2012). Satellite DNA evolution. Genome Dyn..

[24] Garrido-Ramos M.A. (2017). Satellite DNA: An evolving topic. Genes.

[25] Biscotti M.A., Canapa A., Forconi M., Olmo E., Barucca M. (2015). Transcription of tandemly repetitive DNA: Functional roles. Chromosome Res..

[26] Plohl M., Meštrović N., Mravinac B. (2014). Centromere identity from the DNA point of view. Chromosoma.

[27] Bardella V.B., Pita S., Vanzela A.L.L., Galvão C., Panzera F. (2016). Heterochromatin base pair composition and diversification in holocentric chromosomes of kissing bugs (Hemiptera, Reduviidae). Mem. Inst. Oswaldo Cruz.

[28] Pita S., Panzera F., Sánchez A., Panzera Y., Palomeque T., Lorite P. (2014). Distribution and evolution of repeated sequences in genomes of Triatominae (Hemiptera-Reduviidae) inferred from genomic in situ hybridization. PLoS ONE.

[29] Pita S., Lorite P., Vela J., Mora P., Palomeque T., Thi K.P., Panzera F. (2017). Holocentric chromosome evolution in kissing bugs (Hemiptera-Reduviidae-Triatominae): Diversification of repeated sequences. Parasit. Vectors.

[30] Pita S., Panzera F., Sánchez A., Palomeque T., Lorite P. (2017). Chromosome painting in triatomine insects reveals shared sequences between X chromosomes and autosomes. J. Med. Entomol..

[31] Ferree P.M., Prasad S. (2012). How can satellite DNA divergence cause reproductive isolation? Let us count the chromosomal ways. Genet. Res. Int..

[32] Tamura K., Stecher G., Peterson D., Filipski A., Kumar S. (2013). MEGA 6: Molecular evolutionary genetics analysis version 6.0. Mol. Biol. Evol..

[33] Palomeque T., Muñoz-López M., Carrillo J.A., Lorite P. (2005). Characterization and evolutionary dynamics of a complex family of satellite DNA in the leaf beetle *Chrysolina carnifex* (Coleoptera, Chrysomelidae). Chromosome Res..

