# Supplementary material for: Comparative Analysis of Repetitive DNA between the Main Vectors of Chagas Disease: Triatoma infestans and Rhodnius prolixus
Source: Int J Mol Sci. 2018 Apr 24;19(5):1277. doi: 10.3390/ijms19051277 (PMC5983683; doi:10.3390/ijms19051277)
Supplement: Supplementary file 1 [file ijms-19-01277-s001.docx]

10 20 30 40 50 60 70 80 90 100 110 120 130 140 150

....|....|....|....|....|....|....|....|....|....|....|....|....|....|....|....|....|....|....|....|....|....|....|....|....|....|....|....|....|....|

**Rpro-cons** **ATCAAAGGAGTCGTTTTGGAGAAAATCCTTGAAAACTGGAAAATCTATTCAGTAAAGTGGCACTTCCGGAGAGTAGATTTCCACCAAATTTGGGAAATTCAGCCCCACATTTGATTCTTAATAATGTTGTACAGTTGGTAAACCTGTACA**

**1**   **..C.T..A...T........TC..GCA....A..T..................T.T...............T....AC.A............G........**

**2**  **......................................................................................................................................................**

**3**  **.......A..............................................................................................................................................**

**4**  **...........................T.......................A..................................................................................................**

**5**  **...........................T.......................A..................................................................................................**

**6**  **...G......................T........G.A...............................T.G........T...G................AT.T...............T....AC.A.....G.A....G....C...**

160 170 180 190 200 210 220 230 240 250 260 270 280 290

....|....|....|....| ....|... .|....|....|....|....|....|....|....|....|....|....|....|....|....|....|....|....|....|....|....|....|....|....|...

**Rpro-cons**  **ATGTACAGTAATGGCACATG--AGAGGTAA-GTCTAACCACCCCCTCTTGCCCAGAAGTGCCCTTAATTGATTGTTTCCGGGAAAATTTTTCAAATAAAAGTTGTAGAGCTACGTAAGAGCAATTAGCTCACCAAGTTTAAGGGAA**

**1**  **.........G.......T.TTT.A..A...A..........T...-..CA..........................C..........G..A....A...............CT.................................**

**2**  **....................--........-...................................................................................................................**

**3**  **....................--........-................................................................................................T..................**

**4**  **....................--........-...................................................................................................................**

**5**  **....................--........-...................................................................................................................**

**6**  **...................T--........-.........-T......**

**Figure S1**. *Rhodnius prolixus* KQ034390 superconting*.* Alignment of the internal repeats that show similarity with the TinfSat04-1000 satellite DNA of *Triatoma infestans* and consensus sequence derived from them.


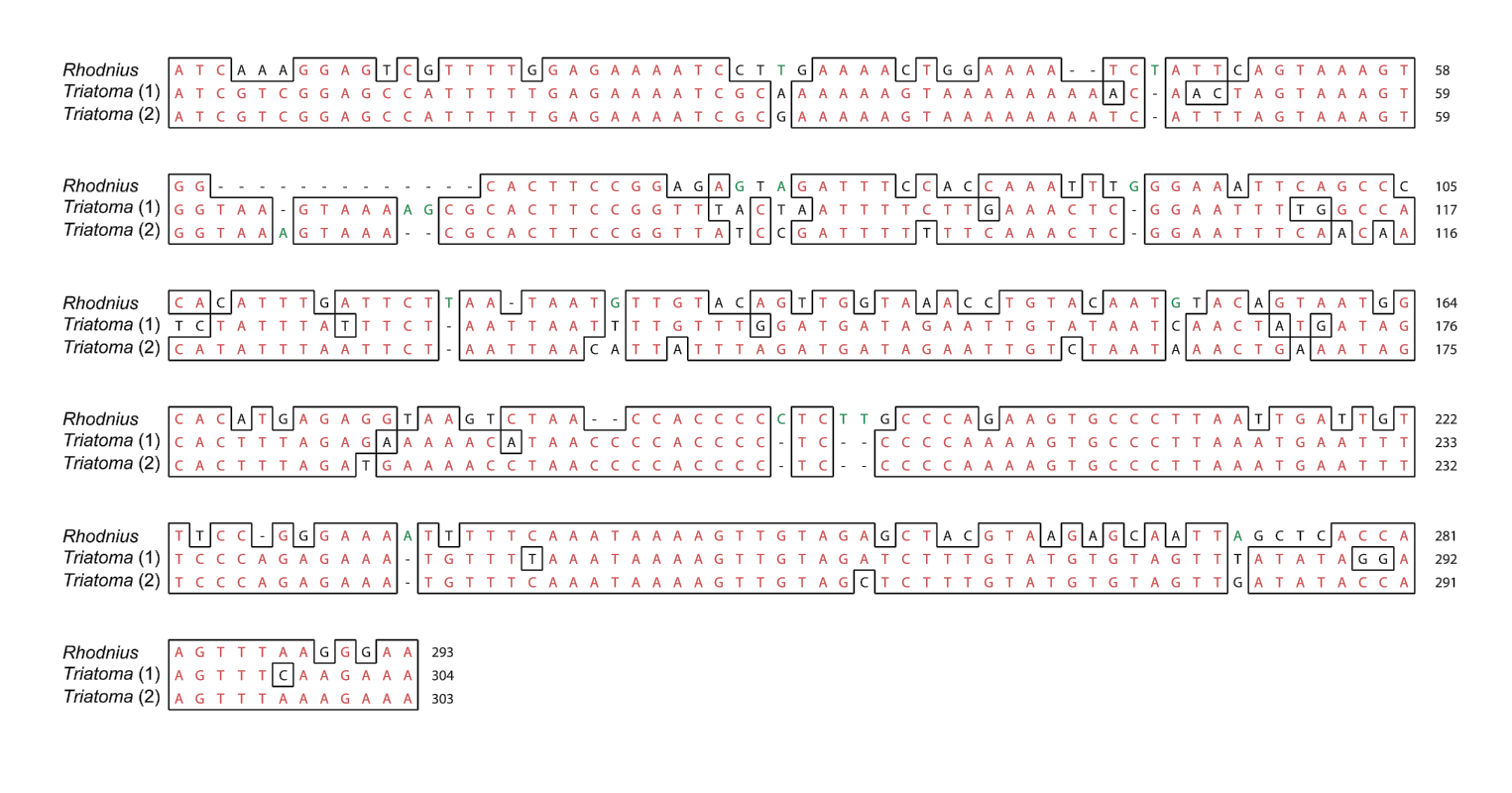


**Figure S2**. Alignment of consensus sequence of the *R. prolixus* repeats (see Figure S1) and the two internal repeats of the TinfSat04-1000 satellite DNA of *Triatoma infestans* (see Figure 4 in the manuscript).

**
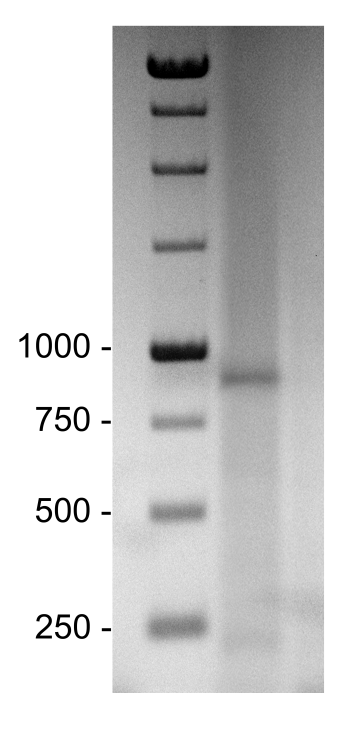
**A B

**
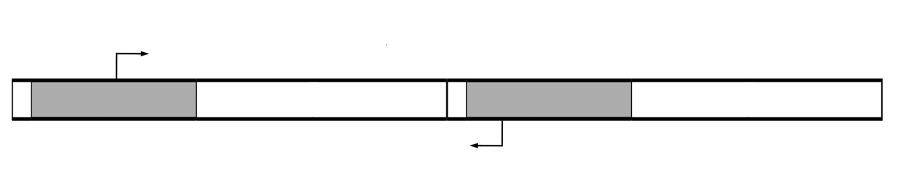
**

C

10 20 30 40 50 60 70 80 90 100 110 120

| | | | | | | | | | | | | | | | | | | | | | | |

**GATATCGAAAATTTGACACG**

**TinfSat04-1000** **GATATCGAAAATTTGACACGATTACAATACTTCCTCTTACTAGGAGTAAGAGAAAGTAAAAAAAAAAAATCTGGTGTGAAACACTCACACAACTTTCTCTTACTCCAGTTCTCAAAATTA**

**Clone-4**  **.................................................A.............------...................................................**

**Clone-14**  **...............................A...T...............C.............----.......................................C...........**

130 140 150 160 170 180 190 200 210 220 230 240

| | | | | | | | | | | | | | | | | | | | | | | |

**TinfSat04-1000** **TAATTGACAAATTTTATAGTTTGTGTACCAAATTTAATGAAATTCGTCGAAAACTGAAAAAAACTGTTCAGTAAAAGCGCACTTCCGGTTTACTAATTTTCTTGAAACTCGGAATTTTGG**

**Clone-4**  **...CAA................T..C..................T........A..T.....-..A....T....G..................G....C...C................**

**Clone-14**  **...........................TG.....A.T.C....A..A...............-.C...T.............A.......AT.......C.......A..T.........**

250 260 270 280 290 300 310 320 330 340 350 360

| | | | | | | | | | | | | | | | | | | | | | | |

**TinfSat04-1000** **CCATCTATTTATTTCTAATTAATTTTGTTT--GGATGATAGAATTGTATAATCAACTATGATAGCACTTTAGAGAAAAACATAACCCCACCCCTCC-CCCAAAAGTGCCCTTAAATGAAT**

**Clone-4**  **.........................CA..C--.........GG.C..........G.......C..........G..GC.G......T..AT.CT.T..T.G..................**

**Clone-14**  **.....C..........G.........T...TT...................................C............G..........T.C..-..T.........A..........**

370 380 390 400 410 420 430 440 450 460 470 480

| | | | | | | | | | | | | | | | | | | | | | | |

**TinfSat04-1000** **TTTCCCAGAGAAATGTTTTAAATAAAAGTTGTAGATCTTTGTATGTGTAGTTTATATAGGAAGTTTCAAGAAAATCGTCGGAGCCATTTTTGAGAAAATCGCGAAAAAGTAAAAAAAATC**

**Clone-4**  **...T..........C.....T.........................T..........GT.T....................G............................G......-..**

**Clone-14**  **...TT....A..........T.............C..A..............G..TC.AC......A...............A......G-...................GG.....-..**

490 500 510 520 530 540 550 560 570 580 590 600

| | | | | | | | | | | | | | | | | | | | | | | |

**TinfSat04-1000** **ATTTAGTAAAGTGGTAAAGTAAACGCACTTCCGGTTATCCGATTTTTTTCAAACTCGGAATTTCAACAACATATTTAATTCTAATTAACATTATTTAGATGATAGAATTGTCTAATAAAC**

**Clone-4**  **.............T....AC..GA.GT...................CC.....T...........................................A....GC.......G........**

**Clone-14**  **.............C.....................C...........A................................................G........G...-.T....T...**

610 620 630 640 650 660 670 680 690 700 710 720

| | | | | | | | | | | | | | | | | | | | | | | |

**TinfSat04-1000** **TGAAATAGCACTTTAGATGAAAACCTAACCCCACCCCTCCCCCAAAAGTGCCCTTAAATGAATTTTCCCAGAGAAATGTTTCAAATAAAAGTTGTAGCTCTTTGTATGTGTAGTTGATAT**

**Clone-4**  **.............C...G.TT...........................................................A..........G......C........A....AA....G.**

**Clone-14**  **............................A.......T.......T..C..........G..................A..........................................**

730 740 750 760 770 780 790 800 810 820 830 840

| | | | | | | | | | | | | | | | | | | | | | | |

**TinfSat04-1000** **ACCAAGTTTAAAGAAAATCGTCGGAGCCATTTTTGAGAAAATCGCAAAAAAGTAAAAAAAAA---CAACTAGTAAAGTGGTACTTCCGGTTGAGGAATTTTGACAGATAACGTACTGTCA**

**Clone-4**  **TAA..................T...................A....................AAA.................T.............................C..A....**

**Clone-14**  **.G....A..............T....T......C.........................TC----....A.........T............................G..CC..C....**

850 860 870 880 890 900 910 920

| | | | | | | | | | | | | | | |

**CGATACGACAAGTGTATGTA**

**TinfSat04-1000** **GATCCCATGGTGATACCTAAACGGAATATCAAGTTTCAACTTTCTACGGTTTTTCGTTTTTGAGCTATGCTGTTCACATACAT**

**Clone-4**  **....G.................................G..................-----.....................**

**Clone-14**  **...G.............................................C.C...............................**

**Figure S3.** (A) Position of the primers Tinf-CL2Uy-F (5' GATATCGAAAATTTGACACG) and Tinf-CL2Uy-R1 (5' ATGTATGTGAACAGCATAGC) used for amplification of the TinfSat04-1000 satellite DNA on *Triatoma infestans*. (B) PCR results showing an amplified band of about 900 bp. (C) Alignment of the sequence of the TinfSat04-1000 satellite DNA with two obtained clones for cloning the PCR product. Sequence in yellow corresponds to the 400 bp insertion (see Figure 4 in the manuscript).

10 20 30 40 50 60 70 80 90

....|....|....|....|....|....|....|....|....|....|....|....|....|....|....|....|....|....|.

**TinfSat12-84** **AAAAAACATATGCGAACACAT---ACAGGCGAGAAGCCATATAAATGTAGTG-AATGTGATTACAGTTGTACACAATCTGGAAATCTT---**

**KQ036412-1**   **...T...G........C..-..........T..CA...ATTT....A.TG..T.A---**

**KQ036412-2**  **...C........AA.......---......A..................CA.-........---...A....CGTGA.AA.T...T.A---**

**KQ036412-3**  **...C.C......A.C.A....---.........................C..-..........T.........ATG..CCATTC.T.A---**

**KQ036412-4**  **...CTT......A........---.........................C..-..........T.........ATGC.CCA.TC.T.A---**

**KQ036412-5**  **...C.C......A.C.A....---......A..................CA.-..........T...G....CGTG..AA.T...T.A---**

**KQ036412-6**  **...C.C......A.C.A....---..T...A..................C..-..........T...A.....ATG..CCA.TC.T.A---**

**KQ036412-7**  **...CG.......A........---T........................CA.-..........T.......A.ATGA.CCA.TC.T.A---**

**KQ036412-8**  **...C.C......A.C.A....---.........................C..-.....A....T.........ATG..CCA.TC.T.A---**

**KQ036412-9**  **...C........A...T....---.........................C..-..........T.........ATG..CCATTC.T.A---**

**KQ036412-10**  **...C.C......A.C.A....---..T...A..................C..-..........T.........ATG..CCA.TC.T.A---**

**KQ036412-11**  **...C........A.C.A....---......A..................C..-..........T...A..T.TAGG.TC.ATTC.T.A---**

**KQ036412-12**  **...CTT......A........---........................GT..-....C..A......A..G....G.ACAT.C..T.A---**

**KQ036412-13**  **...CT.......A....G...---.........................C..-..........T...A....CGTG..AA.T...T.A---**

**KQ036412-14**  **.....C......A........---.........................C..-..........T...A.....ATG..CCA.TC.T.A---**

**KQ036412-15**  **...C.C......A........---T.....A..................C..G..........T........TATG..CCA.TC.T.A---**

**KQ034642-1**   **.-..........T..A......ACG..A....T.T.G---**

**KQ034642-2**  **..G.GG.....A....G....---.........................A..-.....A.C...C.....G..G..C........A.A---**

**KQ034642-3**  **...C.T..........A....---TTTC.G..A..A...C..C......CA.-....C.....T.AAA..GTCACG..CAC.G.AT.G---**

**KQ034642-4**  **.G..C......TTT...G..C---..G....GA.....G.....C.......-.......A..T.TG....GTACT.A...TG.AT.GGGA**

**KQ034642-5**  **.G.C.TAGGCGAA...A....---TTG..A.....A...........C.C..-....C.....T.T.A..G.CAC...CTC.G..T.G---**

**KQ034642-6**  **..G.TC...T...T..AG...---.A......AG.A....T........AA.-.......C..T.T..C..GTTCT..A.ATTTAT.G---**

**KQ034642-7**  **.G..G.....CA....G.A.GCATT...TT.....AA.T..........CCT-T...C..G..T.A.A..TT.TC..T.AATGT.T.G---**

**KQ034642-8**  **...TT......AAA...C...---TT.AAT..T..TA.C.....T.......-..........T.AAAC....A........CT.T.G--**

**KQ034642-9**  **...GC....G.......T...---........A.....T..........AAT-TT........T..G....A.GCG..C...C.AT..G--**

**KQ034642-10**  **-CT.GG..........A....---........A......C............-..................T.G....C..G...T.A---**

**KQ034642-11**  **...C..........C.T....---..T..T..A..A.............C..-....C......T.......CACT.A..C.G..T.A---**

**KQ034642-12**  **.........C.A.T..T....---.A...G...................C..-...........T......G.G.TA.....C.AT.AA--**

**KQ034642-13**  **....G...CG....T......---.........................A..-.T........T.......G.ACT.........A.GA--**

**KQ034642-14**  **...C.......A.T.T.....---.....T..........T..C...C...A-..........T......GT.A....G...C.AT.G---**

**KQ034642-15**  **.G............T.GG..C---.A..........A-..G......AG...-....**

**KQ034383-1**  **....G................---..............T..........C..-....C.....T...ACAG...GT..C.A.C.GT.G---**

**KQ034383-2**  **....T.......T.T.A....---...........A..G.T..TG....T..-....C....TT.....CTT.ACCG.AA.TT..T.A---**

**KQ034383-3**  **....T...CC.A.........---........A.......T..G...C...T-.T..CC...TT..CA...T.AG...CAATG..T.G---**

**KQ034383-4**  **....G.....C.....T....---.........................C..-....C.....T..G..C......CAG.AGTC.T.G---**

**KQ034383-5**  **....CC...............---................TC.....C.CC.-....C.....T..A...G.CAG.AAG.AGC..T.A---**

**KQ034383-6**  **.GG.GC...T.......G...---.....T..A.......T......C.CA.-....C.....T.....C...AG.....ATT....A---**

**KQ034383-7**  **.....C.........G.....CAC..G........A....T......C.C..-.T.......CTG...T.G.TAG..GG.ATC.AT.A---**

**KQ034383-8**  **...TTT........GT.....---..T...A.....----TA.CGA.A.TG.-.G..-A...GT....T**

**Figure S4.** Alignment of the TinfSat12-84 satellite DNA of *T. infestans* with the similar repeats found in the KQ036412, KQ034642 and KQ034383 superconting of *Rhodnius prolixus*.

10 20 30 40 50 60 70 80 90 100

....|....|....|....|....|....|....|....|....|....|....|....|....|....|....|....|....|....|....|....|....

**TinfSat15-99** **TACGTATTGCGTCATACCGTGCAACATGACATGTCTCAACATGTTCATTTTAATTTATTTCTTACTCTTGCTAAAACATCGAAAAAAA-----TACTAGATTTG**

**KQ035712-1**  **-------------------------------------------------------......A..T...C..C.....T.G....T.-------...........**

**KQ035712-2**  **........................................................T...................T..........------....T......**

**KQ035712-3**  **...-----------A.A.........G..A............A........T....................................TAAAA...........**

**KQ035712-4**  **...................G..............................GT...................................------....C......**

**KQ035712-5**  **...-----------A.A.........G..A............A.......ATT---------------------------------------------------**

**KQ034112-1**  **-----------------------------------------------------.C......A..T...C..C.....T.G....T.-------...........**

**KQ034112-2**  **..................................................GT........................T....G.....------....T......**

**KQ034112-3**  **...----------.A..-........G..A............A........T...............................T....TAAAA...........**

**KQ034112-4**  **...................A.....T........................GT...................................------....C......**

**KQ034112-5**  **...----------.A..-........G..A...........---------------------------------------------------------------**

**Figure S5.** Alignment of the TinfSat15-99 satellite DNA of *T. infestans* with the similar repeats found in the KQ035712 and KQ034112 supercontings of *Rhodnius prolixus*.

10 20 30 40 50 60 70 80 90 100 110 120 130 140 150 160 170

....|....|....|....|....|....|....|....|....|....|....|....|....|....|....|....|....|....|....|....|....|....|....|....|....|....|....|....|....|....|....|....|....|....|....|.

**Rhodnius-cons**  **TT-TGGCTTCGTTTGTGCCGGCGATTCACCAAATTTCATCGTTTGTGCCGCATTATTACGCGAGATATTCGCA-------------------------TGTCAAG-TTAGTATGG------------CCACTTT-TTATTTTTTGAGTTTTTTCAAAATTTCTTTTTGCCATTCTT**

**KQ037775-I-1**  **--------------------------------------------------------------------------------------------------------------------------------------------TTTTGAAAAAACTCAAAATTACTTTTTGCCAATCTC**

**KQ037775-I-2**  **TT-TGGCCTCGTTTGTGCCGCTGATTCGCCAAATTTCATCGTTTGTGCCGCATTATTACGCGAGATATTCGCA-------------------------TGTCAAG-TTGGTATGG------------CTACTTT-TTATTTTT-GAGTTTTTTCAAAATTTCTTTTTACCATTCTT**

**KQ037775-I-3**  **TT-AGGCTTCGTTTGTGCCGGCGATTCAACAAGTTTCATCGTTTGTGCCGCATTTTTAGGCGAGATATTCGCA-------------------------TGTCAATATAATTATGACAAAAATTGATATAACTTC-TTATTTTTTGAACTTTTTAAAAATTTCTTTTTGCCAATCTC**

**KQ037775-I-4**  **TT-TGGCCTCGTTTGTGCCGCTGATTCGCCAAATTTCATCGTTTGTGCCGCATTATTACGCGAGATATTCGCA-------------------------TGTCAAG-TTGGTAGGG------------CAACTTT-TTATTTTT-GAGTTTTTTTAAAATTTCTTTTTACCATTCTT**

**KQ037775-I-5**  **TT-AGGCTTCGTTTGTGCCGGCGATTCACCAAGTTTCATCGTTTGTGCCGCATTTTTAGGCGAGATATTCGCA-------------------------TGTCAAT-TTAATATGACAAAAATTGATATAACTTT-TTATTTTTTGAACTTTTTCAAAATTTCTTTTTGCCAATCTC**

**KQ037775-I-6**  **TT-TGGCCTCGTTTGTGCCGCTGATTCGCCAAATTTCATCGTTTGTGCCGCAATATTAAGCGAGATATTCGCA-------------------------TGTCAAT-TTGGTATGG------------CCACTTT-TTATTTTT-GAGTTTTTTCAAAAGTTCTTTTTACCATTCTT**

**KQ037775-I-7**  **TT-AGGCTTCGTTTGTGCCGGCGATTCACCAGGTTTCATCGTTTGTGCCGCATTTTTAGGCGAGATATTCGCT-------------------------TGTCAAG-TTGGAATGG------------CCACTTT-TTATTTTTTCAGTTTTTTCAAAATTTCTTTTTGCCAATCTC**

**KQ037775-I-8**  **TT-TGGCCTCGTTTCTGCTAGCGATTCGCCAAATTTCATCGTTGGTGCCGCATTATTACGCGAGATATTCGCA-------------------------TGTCAAG-TTGGTATGG------------CCACTTT-TTATTTTT-GAGTTTTTTCAAAATTTCTTTTTACCATTCTT**

**KQ037775-I-9**  **TT-AGGCTTCGTTTGTGCCGGCGATTCAACAAGTTTCATCGTTTGTGCCGCATTTTTAGGCGAGATATTCGCA-------------------------TGTCAAT-TAATTATGACAAAAATTGATATAACTTC-TTATTTTTTGAACTTTTTCTAAATTTCTTTTTGCCAATCTC**

**KQ037775-I-10**  **TT-CGGCCTCGTTTGTGCCGCTGATTCGCCCTATTTCATCGTTTGTGCCGCATTATTACGCGAGATATTCGCA-------------------------TGTCAAT-TTAATATGACAAAAATTGATATAACTTT-TTATTTTTTGAACTTTTTCAAAATTTCTTTTTGCCAATCTC**

**KQ037775-I-11**  **TT-TGGCCTCGTTTGTGCCGCTGATTCGCCAAGTTTCATCCGTTGTGCCGCATTATTACGCGATATATTTGCA-------------------------TGTTAAG-TTGGTAGGG------------CCACTTT-TTATTTTT-GAGTTTTTTCAAAATTTCTTTTTACCATTCTT**

**KQ037775-I-12**  **TT-AGGCTTCGTTTGTGCCGGCGATTCACCAAGTTTCATCGTCTGTGCCGCATTTTTAGGCTAGATATTCGCA-------------------------TGTCAAT-TTAATATGACAAAAATTGATATAACTTT-TTATTTTTTGAACTTTTTCAAAATTTCTTTTTGCCAATCTC**

**KQ037775-I-13**  **TT-TGGCCTCGTTTGTGCCGCTGATTCGCCAAATTTCATCGTTTGTGCCGCATTATTACGCGAGATATTCGCA-------------------------TGTCAA------------------------------------------------------------------------**

**KQ037775-II-1**  **--------------------------------------TCGTTTTTGCCGCCACACCAAGCGAGATATTAGCG-------------------------TGTCAAG-TAAATATGAGAAAAATTGATATAACTTT-TCATATTT-GAATTTGTTCAAAATTTCTTTTTGCCAATCTC**

**KQ037775-II-2**  **TT-TGGCCTCGTTTGTGCCGCTGATTCGCCAAGTTTCATCGTTTGTGCCGCATTATTACGCGATATATTTGCA-------------------------TGTTAAG-TTGGTAGGG------------CCACTTT-TTATTTTT-GAGTTTTTTCAAAATTTCTTTTTACCATTCTT**

**KQ037775-II-3**  **TT-AGGCTTCGTTTGTGCCGGCGATTCATTAAGTTTCATCGTTTGTGCCTCATTTTTAGGCGAGATATTCGCA-------------------------TGTCAAT-TTAATATGACAAAAATTGATATAACTTT-TTATTTTTTGAACTTTTTCAAAATTTCTTTTTGCCAATCTC**

**KQ037775-II-4**  **TT-TGGCCTCGTTTGTGCCGCTGATTCGCCAAATTTCATCGTTTGTGCCGCAATATTAAGCGAGATATTCGCA-------------------------TGTCAAT-TTGGTATGG------------CCACTTT-TTATTTTT-CAGTTTTTTCAAAATTTCTTTTTACCATTCTT**

**KQ037775-II-5**  **TT-AGGCTTCGTTTGTGCCGGCGATTCACCAAGTTTCATCGTTTGTGCCGCATTTTTAGGCGAGATATTCGCA-------------------------TGTCAAG-TTGGAATGG------------CCACTTT-TTATTTTTTCAGTTTTTTCAAAATTTCTTTTTGCCAATCTC**

**KQ037775-II-6**  **TT-TGGCCTCGTTTCTGCTAGCGATTCGCCAAATTTCATCGTTTGTGCCGCATTATTACGCGAGATATTCGCA-------------------------TGTCAAG-TTGCTATGG------------CCACTTT-TTATTTTT-GAGTTTTTTCAAAATTTCTTTTTACCATTCTT**

**KQ037775-II-7**  **TT-AGGCTTCTTTTGTGCCGGCGATTCACCAAGTTTCATCGTTTGTGCCGCATTTTTAGGCGAGATATTCGCA-------------------------TGTCAAG-TTGGAATGG------------CCACTTT-TTATTTTTTCAGTTTTTTCAAAATTTCTTTTTGCCAATCTC**

**KQ037775-II-8**  **TT-TGGCCTCGTTTCTGCTAGCGATTCGCCAAATTTCATCGTTTGTGCCGCATTATTACGCGAGATATTCGCA-------------------------TGTCAAG-TTGCTATGG------------CCACTTT-TTATTTTT-GAGTTTTTTCAAAATTTCTTTTTACTATTCTT**

**KQ037775-II-9**  **TT-AGGCTTCTTTTGTGCCGGCGATTCACCAAGTTTCATCGTTTGTGCCGCATTTTTAGACGAGATATTCGCA-------------------------TGTCAAT-TAATTATGACAAAAATTGATATAACTTT-TTATTTTTTGAACTATTTCAAAATTTCTTTTTGCCAATCTC**

**KQ037775-II-10**  **TT-TGGCCTCGTTTGTGCCGCTGATTCGCCAAATTTCATCGTTTGTGCCGCATTATTACGCGAGATATTCGCA-------------------------TGTCAAG-TTGGTATGG------------CCACTTT-TTATTTTT-GGATTTTTTCAAAATTTCTTTTTACCATTCGT**

**KQ037775-II-11**  **TT-GTGCCGCGTTTGTGCCGTCCTTG------------------------------------------------------------------------------------------------------------------------------------------------------**

**KQ034426-I-1**  **---------------------------------------------------------------------------------------------------------------------------------------------------------------------------ATCGT**

**KQ034426-I-2**  **TT-GTGCCGCGTTTGTGCCGGCGATTCACCAAGTTTCATCGTTTGTGCCGCTAAATTACGGAAGTTATTAGCAAAACAAAGTGGCCATACCAACTTG-ACGTCAAGTTAGTATGG-------------CCACCTC-TTTATTTTCAACTTTTTAAAATTTTCTCTGCACCAATCGT**

**KQ034426-I-3**  **TT-GTGCCGCGTTTGTGCCGGCGATTCACCAAATTTTGTCGTTTGTGCCGCTAAATTACGGAAGTTATTAGCAAAACAAAGTGGCCGTACCAACTTGACGTCAAG-TTGGTATGG------------CCACTTT-TTAT-TTTTGAGTTTTTCGAAAATTTCTTCTTACCATTCGT**

**KQ034426-I-4**  **TT-GTGCCGCGTTTGTGCCGGCGATTCACCAAATTTTGTCGTTTGTGCCGCAAAATTACGGAAGTTATTAGCAAAACAAAGTGGCCGCACCAACTTGACGTCAAG-TTGGTATGG------------CCACTTT-TTAT-TTTTAAGTTTTTCGAAAATTTCTTTTTACCATTCGT**

**KQ034426-I-5**  **TT-GTGCCGCGTTTGTGCCGGCGATTCACCAAATTTTGTCGTTTGTGCCGCAAAATTACGGAAGTTATTAGTAAAACAAAGTGGCCGTACCAACTTGACGTCAAG-TTGGTATGG------------CCACTTT-TTATATTTTGAGTTTTTTAAAAATTTCTTTTTACCATTCGT**

**KQ034426-I-6**  **TT-GTGCCGCGTTTGTGCCGGCGATTTACCAAATTT--------------------------------------------------------------------------------------------------------------------------------------------**

**KQ034426-II-1**  **---------------------------------------------------------------------------------------------------GTCAAG-TTGGTATGG------------CCACTTT-TTATTTTT-GAGTTTTTTCAAAATTTCTTTTTACCATTCTT**

**KQ034426-II-2**  **TT-AGGCTTCGTTTGTGCCGGCGATTCACCAAGTTTCATCGTTTGTGCCGCATTTTTAGGCGAGATATTCGCA-------------------------TGTCAAT-TTAATATGACAAAAATTGATATAACTTT-TTATTTTTTGAACTTTTTCAAAATTTCTTTTTGCCAATCTC**

**KQ034426-II-3**  **TA-TGGCCTCGTTTGTGCCGCTGATTCGCCAAATTTCATCGTTCGTGCCGCATTATTACGCGAGATATTCGCA-------------------------TGTCAAG-TTGGTATGG------------CCACTTT-TTATTTTT-GAGTTTTTTCAAAATTTCTTTTTACCATTCGT**

**KQ034426-II-4**  **TT-GTGCCGCGTTTGTGCCGTCCTTTCCCAAAATTTCAGCCATTTAGCTCAAAAATTGAAAAAGTTATTAAGGATTTAAAGTGGCCATACCAACTTGACGGAAGTTGGACAAGCA-------------ATTTCGC-TTGTCTTTGAGATTTTTCAAAATTTTTTTTTGCCATTCCT**

**KQ034426-II-4**  **TT-TGGCCTCGTTTGTGCCGGCGATTCACCAAGTTTCATCGTTTGTGCCGCAATATTAAAGAAGTTATTA----------------------------------------------------------------------------------------------------------**

**KQ034426-III-1**  **----------------------------------------------------------------------------------------------------------------------------------TTTT--CATATTTTGAATTTTTTCAAAATTTCTTTTTGCCAATCTC**

**KQ034426-III-2**  **TT-TGGCCTTGTTTCTGCTAGCGATTCGCCAAATTTCATCGTTTGTGCCGCATTATTACGCGCGATATTCGCA-------------------------TGTCAAG-TTGGTATGG------------CCACTTT-TTATTTTT-GAGTTTTTTCAAAATTTCTTTTTACCATTCTT**

**KQ034426-III-3**  **TT-AGGCTTCGTTTGTGCCGGCGATTCACCAAGTTTCATCGTTTGTGCCGCATTTTTAGGCGAGATATTCGCA-------------------------TGTCAAT-TAATTATGACAAAAATTGATATAACTTT-TTATTTTTTGAACTTTTTCAAAATTTCTTTTTGCCAATCTC**

**KQ034426-III-4**  **TT-TGGCCTCGTTTGTGCCGCTGATTCGCCAAATTTCATCGTTTGTGCCGCATTATTACGCGAGATATTCGCA-------------------------TGTCAAG-TTGGTATGG------------CCACTTT-TTATTTTT-GAGTTTTTTCAAAATTTCTTTTTACCATTCTT**

**KQ034426-III-5**  **TT-AGGCTTCGTTTGCGCCGGCGATTCACCAAGTTTCATCGTTTGTGCCGCATTTTTAGGCGAGATATTCGCA-------------------------TGTCAAT-TTAATATGACAAAAATTGATATAACTTT-TTATTTTTTGAACTTTTTCAAAATTTCTTTTTGCCAATCTC**

**KQ034426-III-6**  **TT-TGGCCTCGTTTGTGCCGCTGATTCGCCAAATTTCATCGTTTGTGCCGCATTATTACGCGAGATATTCGCA-------------------------TGTCAAG-TTGGTATGG------------CCACTTT-TTATTTTT-GAGTTTTTTCAAAATTTCTTTTTACCATTCTT**

**KQ034426-III-7**  **TT-AGACTTCGTTTGTGCCGGCGATTCACCAAGTTTCATCGTTTGTGCCGCATTTTTAGGCGAGATATTCGCA-------------------------TGTCAAG-TTGGAATGG------------CCACTTT-TTATTTTTTGAGTTTTTTCAAAATTTCTTTTTACCATTCGT**

**KQ034426-III-8**  **TT-GTGCCGCGTTTGTGCCGTCCTTTCCCAAAATTTCAGCCATTTAGCTCAAAAATTGAAAAAGTTATTAAGGATTTAAAGTGGCCATACCAACTTGACGGAAGTTGGACAAGCG-------------ATTTCGC-TTGTCTTTGAGATTTTTCAAAATTTTTTTTTGCCATTCCT**

**KQ034426-III-9**  **TT-TGGCCTCGTTTGTGCCGGCGATTCACCAAGTTTCATCGTTTGTGCCGCAATATTAAAGAAGTTATTAAAGAAAAAC-------------------------------------------------------------------------------------------------**

**KQ034426-IV-1**  **--------------------------------------------------------------------------------------------------------------------------------------------TTTTGAATTTTTTCAAAATTTCTTTTTGCCAATCTC**

**KQ034426-IV-2**  **TT-TGGCCTTGTTTCTGCTAGCGATTCGCCAAATTTCATCGTTTGTGCCGAATTATTACGCGCGATATTCGCA-------------------------TGTCAAG-TTGGTATGG------------CCACTTT-TTATTTTT-GAGTTTTTTCAAAATTTCTTTTTACCATTCTT**

**KQ034426-IV-3**  **TT-AGGCTTCGTTTGTGCCGGCGATTCACCAAGTTTCATCGTTTGTGCCGCATTTTTAGGCGAGATATTCGCA-------------------------TGTCAAT-TTAATATTACAAAAATTGATATAACTTT-TCATATTTTGAATTTTTTCA---------------------**

**Figure S6.** *Rhodnius prolixus* KQ034426 and KQ037775 supercontings. Alignment of the internal repeats that show similarity with the TinfSat33-372 satellite DNA of *Triatoma infestans* and consensus sequence derived from them.

**
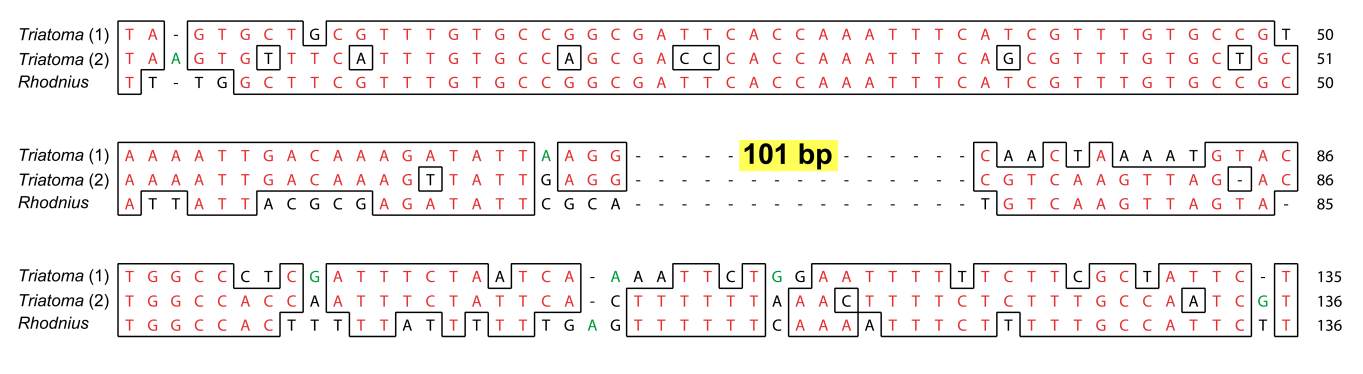
**

**Figure S7.** Alignment of two internal repeats of the TinfSat33-372 satellite DNA of *Triatoma infestans* and the consensus sequence of the *R. prolixus* repeats with similarity with this satellite DNA (see Figure 5 in the manuscript).
